# Improvement in Gender and Transgender Knowledge in University Students Through the Creative Factory Methodology

**DOI:** 10.3389/fpsyg.2020.00367

**Published:** 2020-03-13

**Authors:** Maitane Picaza Gorrotxategi, Naiara Ozamiz-Etxebarria, Eneritz Jiménez-Etxebarria, Jeffrey H. D. Cornelius-White

**Affiliations:** ^1^Department of Didactics and School Organization, Universidad del País Vasco/Euskal Herriko Unibertsitatea UPV/EHU, University of the Basque Country UPV/EHU, Leioa, Spain; ^2^Department of Developmental and Educational Psychology, Universidad del País Vasco/Euskal Herriko Unibertsitatea UPV/EHU, University of the Basque Country UPV/EHU, Leioa, Spain; ^3^Counseling, Leadership, and Special Education, Missouri State University, Springfield, MO, United States

**Keywords:** transgender people, attitudes, sexual education, social education, social change

## Abstract

In Spain, Social Educators, similar to both social workers and educators in the United States, help coordinate social change through educational interventions and mobilization of social groups to benefit marginalized people and overall societal welfare. They are trained to work with diverse populations, and it is important that they have awareness and training on gender and transgender issues given the extensive discrimination that transgender people endue. Research has begun to identify the important role that knowledge and attitudes of health and educational professionals may play in providing a supportive, healing context to combat the harmful effects of this discrimination and how educational trainings may foster improved knowledge and attitudes in helping professions. This study describes a program to improve knowledge and positive attitudes toward gender and especially transgender people in university students who study Social Education. The researchers measured knowledge and attitudes toward gender and transgender people issues of 64 students before and after receiving a 4-month interactive training. They used the Short Form of the Genderism and Transphobia Scale, a 12-item scale of transphobia and gender ideology variables. The researchers also asked participants about their knowledge of gender and transgender issues before and after training. The methodological experience “Creative Factory” was employed as an interactive training program. The main goal of this methodology is to enable students in a formative context to analyze social realities to generate discussion and innovate ideas to design successful practices. After 4 months of training with a weekly session on gender and transgender learning, students showed improvements in knowledge and attitudes toward both gender and transgender people. Specifically, students demonstrated more knowledge about gender and transgender issues and more positive attitudes toward transgender people. The study demonstrates that training in gender education using the Creative Factory methodology improved knowledge and attitudes in students.

## Introduction

The European Union (EU) states in Article 1 of the Charter of Fundamental Rights (2011) that human dignity must be respected and protected. Article 21 of the same Charter censors discrimination on the basis of gender and sexual orientation and in the same year resolution 17/19 recognizes the rights of the LGBT community for the first time including lesbian, gay, bisexual, and transgender people (DePalma and Cebreiro, 2018). Transgender is a general term in which people living their daily lives feel and live as the opposite gender to the one associated with the sex assigned to them at birth (American Psychiatric Association [APA], 2019). The term “transgender” refers to a wide range of social identities and gender presentations (Billard, 2018). In a study conducted in the United States, transgender people were classified into three groups: (1) people who were assigned as men at birth who felt they were women, (2) people who were assigned as women at birth who felt they were men, and (3) those who did not identify as men or women (Factor and Rothblum, 2008). In the last decade in particular, there is growing evidence that, in fact, there is a considerable group of people who do not identify as trans binaries (Motmans et al., 2019).

Transgender people can be subject to severe violence though virtually all are subject to significant and harmful microaggressions and transphobic prejudice (Grant et al., 2011). Transphobia refers to negative beliefs and attitudes about transgender people, including aversion and irrational fear of masculine women, feminine men, transvestites, transgender, or transsexuals (Hill and Willoughby, 2005). The transgender group has historically been a marginalized group, and although today transgender people are more accepted by society, many health and mental health professionals (physicians, psychologists, social educators) either do not have knowledge or positive attitudes and do not believe that they are qualified to provide care services to transgender people and therefore avoid doing so (Kanamori and Cornelius-White, 2016, 2017). There is some evidence that gender expression, perhaps more so than mere sexual orientation or gender identity, may be a factor in the prejudice people experience. While transprejudice is clearly higher among males than females and heterosexuals than LGBT people, there is also evidence that transgender people may be marginalized within the LGBT community when they violate traditional gender roles (Salvati et al., 2018a, b).

It is therefore important for helping professionals who are in contact with transgender people to gain successful educational and training experiences to become familiar with the trans history and culture and demonstrate better interaction patterns with transgender people. It is crucial to design and test interventions with such professionals, preferably early in their training such as during university.

### Literature Review

#### Transphobic Attitudes in General

There is a plethora of research that has been done in the context of gender studies has been on research on sexism and homophobia. And although there are more and more studies that have been conducted on the prejudices that exist against people with transgender identities (e.g., Grant et al., 2011; Morison et al., 2018), comprehensive studies targeting the general transgender population are still lacking (Scandurra et al., 2019). Likewise, there is also budding body of research investigating in particular the attitudes of helping professionals toward transgender persons (e.g., Kanamori et al., 2017; Stryker et al., 2019). While most of these studies are conducted with English-speaking samples, more research is needed with Spanish-speaking samples because they are not many (e.g., Carrera-Fernández et al., 2013) and none specially concerning knowledge and attitudes toward transgender persons within the ranks of social educators.

In a general population study of attitudes toward transgender people with 668 people, the results showed that a majority supported the possibility of transsexuals undergoing sex reassignment; however, 63% thought that the individual should bear the corresponding costs. In addition, a majority supported the right of transgender people to marry in their new sex and their right to work with children. The right of transgender people to adopt and raise children was supported by 43%, while 41% opposed it. The results indicated that those who believe that transsexuality is caused by biological factors had a less restrictive view of transsexuality than people who carry out a psychological view. Men and the older age group were found to have a more restrictive view of these issues than women and the younger age group (Landén and Innala, 2000). This finding has also been found in other studies, where higher scores have been found in men than in women in terms of transphobia (Nagoshi et al., 2008; Norton and Herek, 2013; Elischberger et al., 2016).

As an example of a study concerned with family relations and transphobia, Factor and Rothblum (2008) compared transgender people to their non-transgender siblings, and found that groups of transgender people experienced significantly less social support from their family than their non-transgender siblings. Transgender people also experienced more harassment and discrimination than their non-transgender brothers and sisters.

Another study by Lombardi et al. (2001) investigated the prevalence of transgender people who had experienced violence and discrimination. In their study they found that 60% of the respondents reported being victims of harassment by strangers on the street, verbal abuse, assault with a weapon and/or sexual assault. More than one-third (37%) of respondents also reported being disciplined at work, being degraded or treated unfairly, being fired and, consequently, experiencing economic problems (Hill and Willoughby, 2005).

Although there is evidence that transgender people receive negative attitudes and transphobia from different groups, there are populations in which studies of attitudes toward transgender people demonstrate positive attitudes. Studies with health professionals and feminist communities show that these are populations with generally more positive attitudes toward transgender people (Franzini and Casinelli, 1986; Kendel et al., 1997).

A study by Kanamori and Cornelius-White (2016) showed results consistent with the studies mentioned so far. In their study they found that health professionals in general maintain generally favorable attitudes toward transgender people. The study also found gender differences in attitudes consistent with many previous findings, finding that women showed more accepted attitudes toward transgender people than men.

#### Context of Transphobia in Spain for Educational Interventions

Given the need for studies with Spanish-speaking populations and the site of this study in Spain, this section will review the context that have been carried out on transphobia toward those that identify as transexual thanks to different contributions from activism and academia in this region (Platero, 2014; Platero and Ortega, 2017).

Within the educational framework, different studies confirm the lack of attention to the issue, even though it is an issue that matters to different collectives that work with transgender people. For example, in medicine, where the framework for the interpretation of transsexuality comes from, the National Centre for LGBT Health Education offers educational programmes, resources and consultations to health care organizations with the aim of optimizing quality and cost-effective medical care for lesbian, gay, bisexual, and transgender (LGBT) people (National LGBT Health Education Center, 2019).

Another organization for the visibility of transsexual minors is the appearance of the Association of Families of Transsexual Minors, *Chrysallis*, which is fighting for society, health and schools to meet the needs of transsexual children on an equal footing with cisexual children. To this end, they have made a list of about seventy schools they call transfriendly to facilitate the path of minors. Among the educational needs of the minors the association points out the essential “The training of all personnel related to the educational process, teachers, counselors, psychologists, assistants, social workers and management teams, as well as the training of students” (Gavilán, 2015, p. 85).

These examples reflect current social change in the interpretation of gender and sexuality. However, much remains to be done for these people to freely develop their identities. Various researches and studies indicate that, in the field of formal education, there are no training programs, and gender diversity is an issue that is not contemplated when different studies detect the need to work with students. For example, the European Union Agency for Fundamental Rigths [FRA], 2013), produced the largest set of empirical information with the LGBT collective to date with 93,000 people over 18 years of age in the EU, where its highlighted that members of this community can not be themselves in their daily lives. The results showed the following data: 47% of respondents had felt discriminated against or harassed because of their sexual orientation; more than 80% remembered negative comments or acts of bullying in the school environment and 67% of respondents stated that they hid their sexual orientation in the school stage.

In Spain, homophobic bullying has always been present in schools. INJUVE (2011) stressed that the homophobic collective imposes its law in classrooms in the face of the passivity of other students and teachers. In this line, some authors highlight the importance of the role of the observer as a facilitator of abuse (Gini, 2006; Byers, 2013). A little later, in 2012/13, the Education Commission of the Lesbian, Gay, Transsexual and Bisexual Collective of Madrid (COGAM) together with the State Federation of Lesbians, Gays, Transsexuals and Bisexuals (FELGTB) carried out a study on sexual diversity in the classrooms where the results showed that of 653 children under 25 years of age who acknowledged having suffered bullying because of sexual orientation, 43% had come to devise suicide highlighting the failure of the school system (DePalma and Cebreiro, 2018). Against this backdrop, Pichardo and Puche (2019) decide to focus on the attitude and practices of teachers in the face of sexual diversity. The results show that nursery, primary and secondary school teachers think that not being heterosexual or skipping gender traits or traits related to appearance are the reasons that generate the most insults or rejection. As we have read in the previous point, gender is also a variable where men are more likely to insult and less likely to ask for help and women are more likely to face issues of diversity and coexistence in the classroom. Finally, there is a constant demand for training on the part of the actors involved, both for teachers and students, since both groups are victims of discrimination (insults, mockery, exclusion) due to their personal characteristics (Pichardo and Puche, 2019).

In this path of discrimination prior to the university stage, schools do not guarantee measures against the stigmatization and marginalization of these people where the educational dimension of heterosexual and patriarchal norms continues (Elipe et al., 2017; Alegre, 2018). In the universities the panorama is not better either, the forms of identity and the new considerations associated to the inclusion of sexual diversity continue being a pending subject due to the strong cultural roots and the gender binarism. In addition, the concept of university is historical and maintains its essence, its raison d’être transcends all time, place or social circumstance without reforms prevailing (Medina, 2005). Proof of this is that despite the fact that different media such as literature, cinema, plastic and audiovisual arts or advertising have introduced transsexual experiences in the educational sphere, the same does not happen in the academic sphere where there is a generalized misinformation about the LGBT+ community (Castro and Ramos, 2019). Basque (Ley 09/2019, 2012), of 29 June^1^ (Spain), includes in its articles 16 and 17 the obligation to incorporate methods, curricula and educational resources that serve to increase understanding and respect for the diversity of gender identities by dictating actions in matters of transsexuality. However, this law only works for basic education reflecting university absence.

Faced with this panorama, the university responds to heternormativity that is structured in a dichotomous system of male-fall-masculine and female-vulva-feminine. Therefore, LGBT people continue to be constructed as minorities respecting a community of equals made up of heterosexual people. This means that they are conceptualized from the discourse of otherness and from a hegemonic and heteronormative position. What generates that the educational intervention reproduces discourses that consider these people as deficit, limiting them in agency (Galaz et al., 2016).

Faced with this situation, trans people exclude themselves, when choosing university studies they opt for training spaces perceived as safer and more respectful such as careers related to art, feminized (teaching or nursing) and humanities studies and related to social change also attracts them. However, more masculinized careers such as engineering or scientific-technical ones perceive them as less desirable. Despite the fact that in some universities there are associations of LGBT students, in general, the university is created as an androcentric and eurocentric space that strips itself of affectivity and focuses on science. Thus, the university has become a space full of physical, bureaucratic and symbolic barriers for LGBT people (Pichardo and Puche, 2019).

### The Creative Factory Intervention

For several years, the El Observatorio del Tercer Sector de Bizkaia (OTSB) (Fundación EDE, 2016) has been developing the creative factory (CF) methodology as an educational intervention that generates reflections and innovative solutions to significant social problems and which aims to generate interaction between different agents. This proposal was born from the meeting of people and collectives working for social transformation from multiple fields, such as that of unaccompanied immigrants, people in processes of exclusion and with severe mental illness, mistreatment among peers or the response to violent behavior in adolescents, among others. In this way, students reflect from the critical (social, political, systemic) to foster creativity in order to respond to the integral development of the personality and to ensure that the educational institution is not content with merely reproducing the social system, but fulfils its function of transforming reality and that future professionals can develop alternative strategies to respond to social demands (Rodrigo and Rodrigo, 2012). In addition, making use of creativity, professionals are able to adapt to new changing contexts and can contribute significantly to society (Goñi, 2000; Chacón and Moncada, 2006). A recent study studying the creativity of university students concludes that students show greater creativity after having fostered it in class (Caballero et al., 2019).

The CF methodology has been applied since the 2011/2012 academic year in the subject of General Didactics. We have based and been inspired by the process carried out by Alonso and Arandia (2014) but adapting it to the current group and making modifications. On this occasion, we introduce a growing topic relating to transsexual persons, adapting the methodology to the needs of the students after evaluation (2018/2019) for continuous improvement. Although the subject of transgender people has been introduced throughout the continuous assessment, the methodology of the CF is carried out through training sessions consist three seminars. That is to say, in order to deepen the theme and offer more formation, the CF process is accompanied by different interventions and educational activities throughout the 4-month period (September–December).

### Objective and Hypothesis

Social educators work in many areas, with different vulnerable populations, including transgender populations. For this reason, the importance of training these professionals so that they can act and intervene in educational spaces as well as in family, work and community spaces is highlighted (Parcerisa-Aran and Forés, 2003; Bas-Peña et al., 2014).

It is therefore important to assess the level of knowledge on gender and transgender issues in Social Education students and to design educational models that train students in these issues. In addition, it is also important to know the attitudes that they have toward transgender people since many times negative attitudes or concrete stereotypes are given from ignorance. Education is one of the basic tools for students to get to know this group and improve their knowledge and attitudes.

The main objectives of this study were, on the one hand, to measure the attitudes of social education students toward gender and transgender people, and on the other hand, to value the knowledge about transgender people in these students. And finally, to measure the changes in attitudes and knowledge after an education program in transgender people based on the creative factory methodology.

Hypotheses suggest that Social Education students would have positive attitudes toward gender and transgender people prior to taking the course. Since students do not receive much information on gender and transgender issues during their university studies, in terms of knowledge, it is expected that students will not have much knowledge on the subject of transgender before taking the transgender training course. As other studies have shown, women are expected to have better attitudes than men toward transgender people. Finally, thanks to the creative factory methodology, improvements are expected in both knowledge and attitudes toward gender and transgender people.

## Materials and Methods

### Participants

The sample was taken from second-year Social Education degree students in the Public University of the Basque Country (Leioa, Spain). The researchers offered them voluntary participation in this study. 64 people participated in the study. The average age of the subjects was 20.23 years. 81% (52 people) of the participants were women, 17% (11 people) were men and 2% (1 person) was not identified as either men or women.

All the students participated on a voluntary basis, received information about the procedure of the investigation and gave their consent before participating in the study. Therefore, the procedure followed is approved by the Ethics Committee respecting the Helsinki Declaration of the World Medical Association.

### Measures and Instruments

#### The Short Version of the Gender and Transphobia Scale

As Billard (2018) says, so far, there are six published scales for measuring attitudes toward transgender people: the Gender and Transphobia Scale (GTS; Hill and Willoughby, 2005), Transphobia Scale (TS; Nagoshi et al., 2008), Transgender Attitudes Scale (ATTI; Walch et al., 2012), Transgender Attitudes and Beliefs Scale (TABS; Kanamori et al., 2017), Transgender Prejudice Scale (Case and Stewart, 2013) and Transprejudice Scale (for transgender women; Winter et al., 2009).

The Gender and Transphobia scale is a scale developed and validated in Canada (GTS; Hill and Willoughby, 2005) and analyses negative attitudes toward trans people, including transsexuals, transgender, and transvestites. It assesses the cognitive (gender), affective (transphobia) and behavioral (gender attack) of the co-components mentioned. It is a scale that has been translated and validated in several cultures.

The scale used in this study is the short version of the GTS was validated in Spanish with a stable factor structure and adequate reliability (Carrera-Fernández et al., 2013). The brevity of the instrument saves time and increases the effectiveness of the evaluation processes. It is a test with good psychometric properties. The Cronbach’s alpha of their corresponding subscale indicated good psychometric properties. The scale showed good reliability, with a α = 0.80 for Gender Bashing and a α = 0.83 for Transphobia = Genderism (Carrera-Fernández et al., 2013).

The scale is a 12-item scale that measures the variables of Gender Bashing, transphobia and genderism. The genderism is a belief system based on a heteronormative social model. Genderism devalues people who do not adjust to their gender roles or whose sex is not consistent with their gender. The transphobia is the attitudinal component and this includes negative feelings, aversion and fear of people who transgress the rigid two-gender model. Gender bashing is the act of victimizing a person emotionally, physically, sexually, or verbally because they are transgender. It is the behavioral component of sexism (Carrera-Fernández et al., 2013).

The first six items of the short version of the GTS measures gender bashing and the last six transphobia and genderism. The answers are answered on a scale from 1 to 7 with the following values: 1 is strongly agree, 2 agree, 3 somewhat agree, 4 neutral, 5 somewhat disagree, 6 disagree and 7 Strongly disagree. Lower scores indicate a higher level of transphobic attitudes. The lowest score that can be obtained in these two factors would be a 7, indicating high levels of gender bashing and transphobia/genderism. The highest score that can be obtained in these two factors would be a 42, indicating absence of gender bashing and transphobia/genderism.

#### Scale to Measure Transgender Knowledge and Other Variables

For clarity and ease of administration, a single item measure employing Llikert scale of 1 to 10 was used to self-assess students’ knowledge of transgender people. Students had to evaluate their knowledge about transgender people: 1 being a complete lack of knowledge about the subject, and a 10 an optimal knowledge about the subject. The item was: my level of knowledge about what it means to be a transgender person is. The Likert scale places each individual at a particular point of knowledge. This scale is used to help the respondent assess his or her knowledge about the topic being asked. It allows us to measure the degree of knowledge that the respondent considers to have regarding a specific topic (Ospina et al., 2005). Other variables collected were the age and gender of the students answering the questionnaire. It was also asked if they personally know any people who are transgender.

### Procedure

The first step was to secure permission from the university ethics committee to carry out this research. The project took place in a Spanish University with a World ranking in the top 500 universities within the undergraduate program of Social Education, which is composed of seven modules. Specifically, we are located in the subject of General Didactics belonging to the third module called Foundation of Educational Processes, which is taught in the first 4-month period of the second-year (2019/2020).

On the first day of class the students were informed about the study, and the people who decided to voluntarily participate in the study completed the pre-tests using the google forms platform. The questionnaires answered by the students were anonymous and had to include a code in order to identify the relationship between the questionnaires carried out before and after the educational intervention.

After the training they retook the measures again.

### Data Analysis

The data of the participants were collected through google forms. To begin with, descriptive analyses were carried out for sociodemographic data, transgender knowledge, and Short Version of the GTS results. Paired *t*-test for related samples were calculated to compare the means between the test and retests in the variables of knowledge about transgenderism, and the gender bashing and transphobia/genderism factors of the GTS. *t*-tests were also performed for independent samples to see differences in Short version Gender and Transphobia Scale questionnaire factors between men and women. To analyze the data we used the program R-comander program and the results were reflected in tables.

## Results

Table 1 shows different descriptive variables of the sample, including maximums, minimums, means, and number of people and percentages of men, women and non-binary persons among the participants. The data show that the average age of the participants was 20.23 years and that most of the participants were women (81%).

**TABLE 1:** Age, gender, knowledge of any transgender person and knowledge of transgender issues of the study participants.

**Age**	**Mean**		**Maximum**		**Minimum**	
	20.23		29		18	

**Gender**	**Women**		**Men**		**Other**	
	***N***	**%**	***N***	**%**	***N***	**%**
	
	52	81	11	17	1	2

**Know a transgender person**	**No**		**Yes, but not personally**		**Yes, a close person**	
	***N***	**%**	***N***	**%**	***N***	**%**
	
	17	27	31	48	16	25

**Knowledge of transgender issues on a scale of 1 to 10**	**Less than 3**		**Between 3 and 7**		**More than 8**	
	***N***	**%**	***N***	**%**	***N***	**%**

	3	5	39	61	22	34

Knowledge about transgender issues is divided between groups that have scored less than 3, from 3 to 7, and more than 8 on a scale of 1 to 10. Where 1 would be the minimum knowledge about transgender topics, and 10 would be the optimal knowledge about transgender topics. Finally Table 1 includes the number of people and the percentage of people who had close relationships with transgender people, had acquaintances (not close relationships), and those who did not know transgender people. Most of the participants knew a transgendered person, although not necessarily (48%), and knowledge about transgender people was medium in most participants (61%).

Table 2 shows the comparison of means of knowledge about transgender people, gender bashing, and transphobia/genderism of the Short version Gender and Transphobia Scale between the test and the retest according to the *t*-test for related samples. The data show that there was a statistically significant improvement in knowledge about transgender. In the gender bashing and transphobia dimensions there were improvements although not significant.

**TABLE 2:** *t*-test for comparison knowledge about transgender people, gender bashing, and transphobia/genderism before and after training.

Paired *t*-test	Pre mean	Pre SD	Post mean	Post SD	*p*-Value
**Level of transgender knowledge** (Scale from 1 to 7, Higher scores indicate a higher level of knowledge)	6.76	1.61	7.29	1.39	0.015
**Gender bashing total factor** (Scale from 1 to 7, Lower scores indicate a higher level of transphobic attitudes)	40.58	3.39	40.36	3.23	0.219
**Transphobia/genderism total factor** (Scale from 1 to 7, Lower scores indicate a higher level of transphobic attitudes)	40.90	3.27	41.42	1.73	0.208

The mean comparisons shown in Table 3 were made between people who defined themselves as women or men. The differences between men and women in gender bashing were significant, with men having more gender bashing. There were no significant differences in transphobia. There was one person who did not define himself as either a man or a woman. But being only one is not representative to make a comparison of means between different genders. Therefore we will describe below the characteristics of this person. The scores in gender bashing was 42 and in transphobia 42 being the highest scores that can be taken on this scale and representing a very low level of both factors in this person.

**TABLE 3:** *t*-test for the comparison of independent means between men and women of the factors of gender bashing and transphobia/gender.

	Men mean	Men SD	Women mean	Women SD	*p*-Value
**Gender bashing**	36.54545	6.817091	41.40385	0.9550611	0.039
**Transphobia/genderism**	38.90909	5.769827	41.30769	2.380793	0.203

*Lower scores indicate a higher level of transphobic attitudes.*

## Discussion

The descriptive data show the characteristic data of the students of Social Education where the average age is around 20 years old and the great majority of people are women. There was only one person who was not considered binary (neither male nor female).

Throughout the study, the relevance of making the reality of transgender people known has been justified in order to put an end to discriminatory attitudes and behaviors. Moreover, in social education professionals whose socio-educational work promotes the achievement of social change (Parcerisa-Aran and Forés, 2003; Bas-Peña et al., 2014). The following is a review of the results found and the explanations that justify these results with the review of the scientific literature.

The first objective of the study was to explore the level of knowledge that students of the social education degree perceive to have toward what it means to be a transgender person. In this research, only 34% believe they have optimal knowledge. Therefore, the hypothesis is fulfilled that the students would not have much knowledge about this subject before having received specific training. The previous evidence showed the lack of information about LGBT+ collectives in the academic field (Castro and Ramos, 2019). As well as professionals in contact with transgender people, with a perception of positive attitudes toward them, they did not feel qualified to respond to their needs due to the lack of training. Other research stated that trans people when choosing university studies could be inclined toward degrees related to social change or the humanities, perceiving them as more respectful and, therefore, safe environments (Pichardo and Puche, 2019). In our study, dealing with a humanities degree, it has been found that only 25% of the participants say they know a transgender person closely, while 27% say they do not know any transgender person. This underlines the importance of increasing knowledge about this group even in those professions in which there is a greater sensitivity to work with disadvantaged groups (Gavilán, 2015).

Several studies have found that transgender people experience violence and discrimination (Lombardi et al., 2001; Hill and Willoughby, 2005). Fortunately, there are populations such as health professionals and feminist communities that have positive attitudes toward transgender people (Franzini and Casinelli, 1986; Kendel et al., 1997). As has been observed in this study, social educators are also a population with positive attitudes toward this group. Considering that they are professionals who work actively in different social contexts, their training on gender and transgender issues is important (Gavilán, 2015, p. 85).

The second objective was to analyze the attitude of Social Education students toward transgender people. According to the hypothesis of the study, it was expected to find positive attitudes toward transgender people because of the sensitivity or respect that is expected of students in the degree of social education toward disadvantaged groups. In this case, a fairly low transphobia and gender aggressiveness score was found, which is why this hypothesis was affirmed. Also in a study with a sample of 668 people, positive attitudes toward transgender people were found, such as the recognition of the right to adoption, among others (Landén and Innala, 2000). Fortunately, there are populations such as health professionals and feminist communities that have positive attitudes toward transgender people (Franzini and Casinelli, 1986; Kendel et al., 1997). From this study it can be deduced that the Social Education student body is also a population with positive attitudes toward this group. Regarding negative attitudes, several studies have found that transgender people experience violence and discrimination (Lombardi et al., 2001; Hill and Willoughby, 2005), which prevents them from being able to behave according to their identity because of the inadequate treatment they received (Hill and Willoughby, 2005; European Union Agency for Fundamental Rigths [FRA], 2013). This research shows that 85 and 71%, respectively, of the mockery that has been directed at women for showing a male aspect or behavior or at men for their female aspect or behavior, state that they have not made any mockery and 92% have not behaved violently toward women for their male behavior or toward men for their female behavior. Aversion or fear of transgender people (e.g., male women and female men) are attitudes that are part of transphobia (Hill and Willoughby, 2005) and need to be eliminated.

In terms of gender differences, the results of this study show that men have lower scores than women on gender bashing and transphobia/genderism. Despite a small sample of men compared to women, men showed significantly more discriminatory responses than women on the gender bashing scale. The results also suggest there may be more transphobia in men than in women although the results are not statistically significant. These findings coincide with other studies showing that men have more transphobia than women (Landén and Innala, 2000; Nagoshi et al., 2008; Norton and Herek, 2013; Elischberger et al., 2016; Kanamori and Cornelius-White, 2016).

In reference to the third objective, the aim was to study the changes given in attitude and knowledge in the students of Social Education after receiving training on the transgender subject. It was expected to find an improvement after the training through the creative factory methodology. This hypothesis has been partially fulfilled, given that no differences have been collected in the improvement of attitudes toward the collective; one explanation may be that from the beginning the average of transphobia and gender aggressiveness found was low and although in transphobia an improvement is observed, this has not turned out to be statistically significant. Range restriction (the mean was already very high on the scale, indicating low transphobia) may account for the finding, suggesting that future studies should use measures with a wider range that may be more sensitive to change.

On the other hand, there has been a statistically significant difference in the perceived knowledge on the subject of transgender, having increased the knowledge after receiving the training, so it can be stated that the training received has made it possible for the participating students to increase their knowledge. In a previous investigation with students and teachers in the field of health, it was found that after a training of 8 h the knowledge, attitudes and clinical preparation toward people of sexual and gender minorities improved with respect to the control group that had not received any training (Pratt-Chapman and Phillips, 2019). Thus, learning programs on transgender issues improve both knowledge and attitudes toward transgender people. For this reason, the importance of promoting training courses on gender and transgender for professionals so that they can act and intervene both in educational spaces and in family, work and community spaces (Parcerisa-Aran and Forés, 2003; Bas-Peña et al., 2014) is highlighted.

This training program on gender and transgender has created a context of reflection and knowledge generation for students using the creative factory methodology (OTS, 2016). This methodology makes use of creativity and, thanks to this, facilitates the capacity to adapt to new changing contexts and can contribute significantly to society (Goñi, 2000; Chacón and Moncada, 2006). In this study, it has been demonstrated that through the creative factory methodology, changes can be achieved both in attitudes and in the students’ knowledge about gender and transgender issues. This demonstrates that the methodology has served to improve knowledge on transgender issues.

The current study is subject to several limitations. The use of a single item measure to measure the students’ perception of transgender knowledge is one obvious drawback as reliability and validity information are not available for the use of this measure. The lack of a control group and the small sample size for a quantitative study offer further constraints for the internal and external validity of the study. Future research could employ more validated measures, comparison groups using no intervention or other interventions to compare effectiveness and larger, more diverse Spanish speaking sample sizes. Future lines of research also aim to collect information from university students of different grades. In this way, it will be possible to carry out a comparative analysis between students from different disciplines. Another future line of research is to carry out a qualitative study where the results are focused on the innovative contributions of the students. In this case, an analysis of the good practices and innovative ideas presented by the students will be carried out.

As mentioned above, despite the importance of gender training for Social Education students, studies show that they receive little training on the subject (Bas-Peña et al., 2014). An objective for future studies is to continue creating this type of training both at the Social Education level and at other levels for which it is even more necessary to develop skills in relation to the relationship and treatment with people, in order to continue promoting awareness and learning about gender and transgender issues.

## Data Availability Statement

The datasets generated for this study are available on request to the corresponding author.

## Ethics Statement

The studies involving human participants were reviewed and approved by Ethics Committee for Research Related to Human Beings (CEISH) of the University of the Basque Country. The patients/participants provided their written informed consent to participate in this study.

## Author Contributions

MG, NO-E, and EJ-E were involved in the conceptualization of the project and involved in the acquisition of data and analysis. MG, NO-E, EJ-E, and JC-W were involved in the interpretation of the data. All authors were involved in the drafting and revising of the work for intellectual content, provided approval for submission for publication of the content, and agreed to be accountable for the accuracy and integrity of the project.

## Conflict of Interest

The authors declare that the research was conducted in the absence of any commercial or financial relationships that could be construed as a potential conflict of interest.

## Footnotes

1 LEY 09/2019, de 29 de junio, de no discriminación por motivos de identidad de género y de reconocimiento de los derechos de las personas transexuales. (accessed October 24, 2019)
